# Supplementary material for: Thrombocytosis and Effects of IL-6 Knock-Out in a Colitis-Associated Cancer Model
Source: Int J Mol Sci. 2020 Aug 27;21(17):6218. doi: 10.3390/ijms21176218 (PMC7504541; doi:10.3390/ijms21176218)
Supplement: Supplementary file 1 [file ijms-21-06218-s001.zip › ijms-874497 supplementary/Uploading supplementary/Fig S5.pdf]

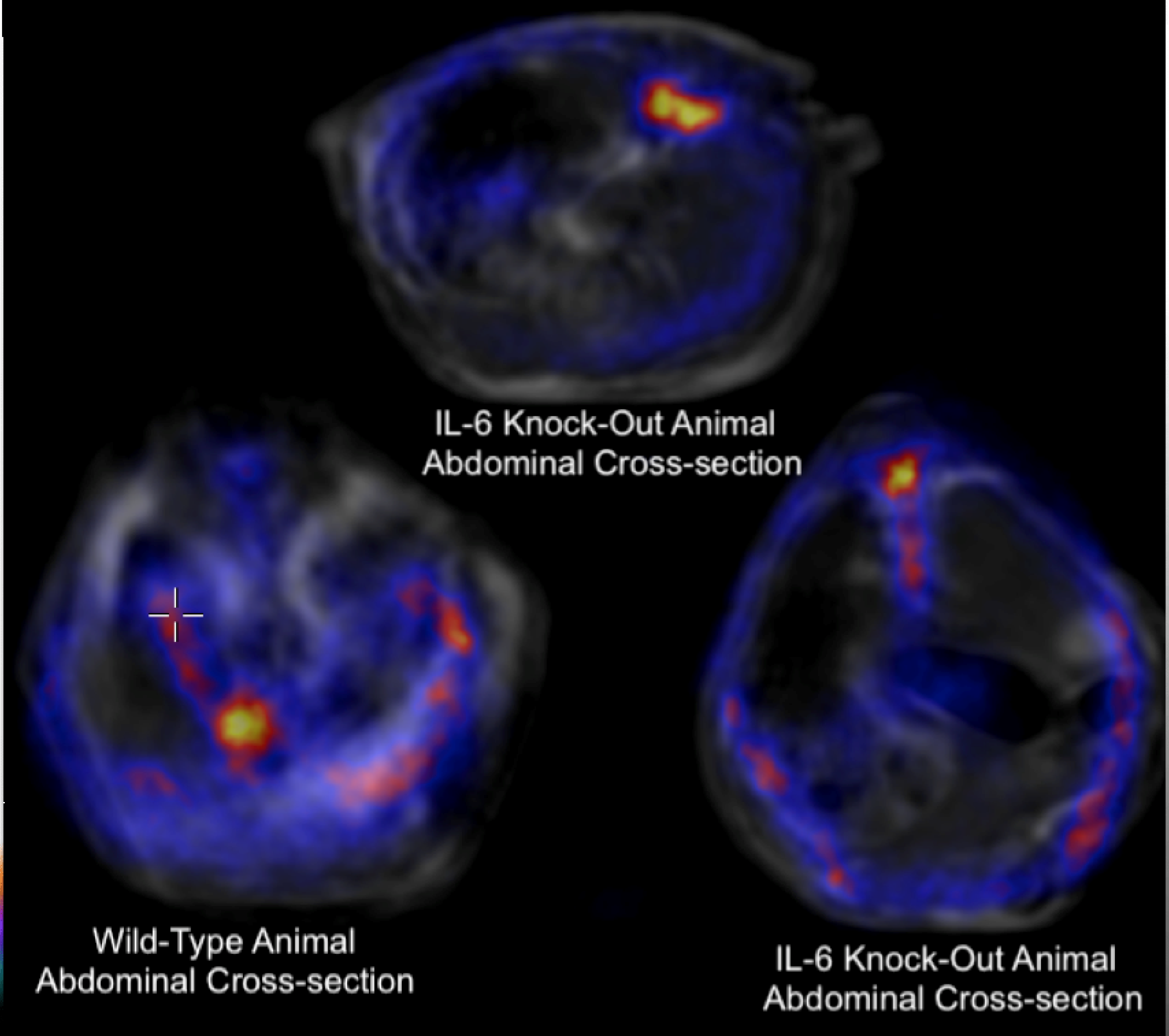
IL-6 Knock-Out Animal
Abdominal Cross-section
Wild-Type Animal
Abdominal Cross-section
IL-6 Knock-Out Animal
Abdominal Cross-section
