# Supplementary material for: Thrombocytosis and Effects of IL-6 Knock-Out in a Colitis-Associated Cancer Model
Source: Int J Mol Sci. 2020 Aug 27;21(17):6218. doi: 10.3390/ijms21176218 (PMC7504541; doi:10.3390/ijms21176218)
Supplement: Supplementary file 1 [file ijms-21-06218-s001.zip › ijms-874497 supplementary/Uploading supplementary/Supplementary information_Proofread.docx]

**Supplementary Data for the Manuscript entitled:**

**Thrombocytosis and effects of IL-6 knock-out in a colitis-associated cancer model**

**IL-6 gene knockout**

The *IL-6* KO strain of mice was established using a homologue template. We opted to modify a few base pairs only by inserting a STOP codon. This ensures the necessarily high efficiency of the CRISPR/Cas9 targeted gene editing mechanism of the *IL-6* gene. Also, we applied a higher efficiency oligonucleotide donor to modify the gene and insert a STOP codon.

In order to ensure that all transcript variants with alternative splicing of the IL-6 protein can be stopped, we needed to select a KO strategy encompassing all variants.

Therefore the STOP codon was decided to be placed downstream from the alternative start codon on the IL-6 gene. We have inserted a TAAxTAG sequence to the correct frame. Subsequent sequening of the region has confirmed the correct insertion of this codon.

Figure presents the exons targeted, position: Chromosome 5, 30,013,114-30,019,981, ‚forward strand’. The alternative start codons are underlined.

Second exon of variant 1,2, and 3.

GAGACTTCCATCCAGTTGCCTTCTTGGGACTG*ATG*CTGGTGACAACCACGGCCTTCCCTA CTTCACAAGTCCGGAGAGGAGACTTCACAGAGGATACCACTCCCAACAGACCTGTCTATA CCACTTCACAAGTCGGAGGCTTAATTACACATGTTCTCTGGGAAATCGTGGAAATGAGAA

AAGAG

First exon of variant 4:

AGGCGCCCAACTGTGCTATCTGCTCACTTGCCGGTTTTCCCTTTTCTCCACGCAGGAGAC TTCCATCCAGTTGCCTTCTTGGGACTG**ATG**CTGGTGACAACCACGGCCTTCCCTACTTCA CAAGTCCGGAGAGGAGACTTCACAG**AGG**ATACCACTCCCAACAGACCTGTCTATACCACT TCACAAGTCGGAGGCTTAATTACACATGTTCTCTGGGAAATCGTGGAAATGAGAAAAGAG

Data were obtained from the Ensembl data base.

*IL-6* gene knockout was confirmed by lipopolysaccharide (LPS) stimulation. LPS is an endotoxin present in the cell wall of Gram-negative bacteria; it induces IL-6 production when acting on – among others – macrophages. During the confirmation of the knockout, the mice were treated with either physiological saline or LPS (2.5 mg/kg BW) dissolved in saline, both administered by the intra-peritoneal route. The presence of IL-6 was detected by ELISA assay.

*Figure S1**:* *LPS induced IL-6 plasma concentrations (mol/L) measured in the individual groups with ELISA.* WT and *IL-6* KO animals were administered saline or LPS by intraperitoneal injection. Naïve animals were not treated. Data represent the mean (SD) *** *P* < 0.001.


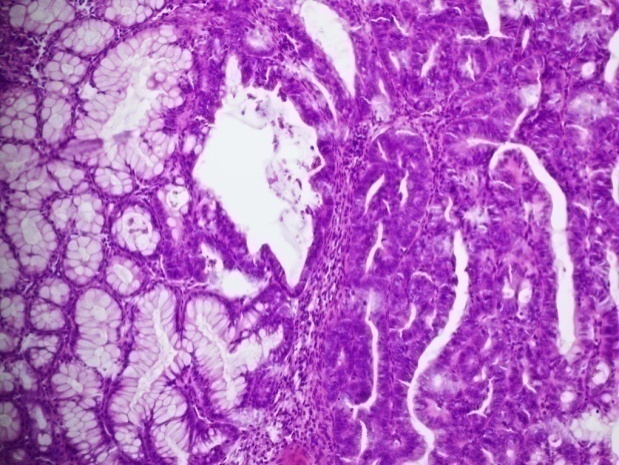


*Figure S2:* *Histology of pTis colon carcinoma in a WT mouse colon (H&E staining, 100x magnification).* Microphoto of a lesion of the large bowel mucosa with normal glands (on the left), and with glands exhibiting neoplastic transformation (on the right).


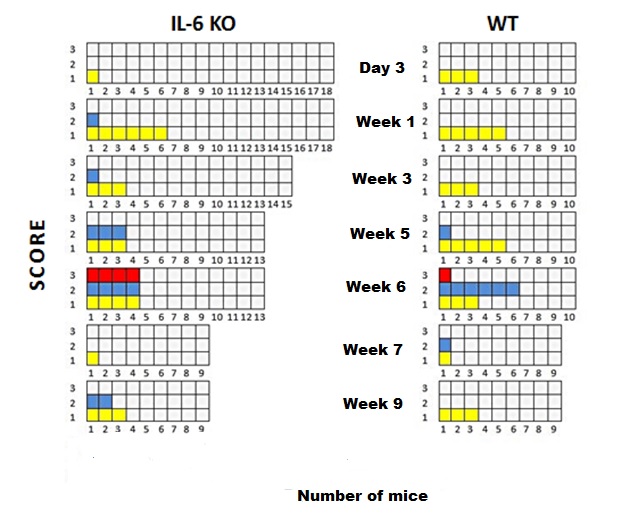


*Figure S3: Decimal scoring system to measure the severity of inflammation.* White – normal, yellow – watery stool/mushy stool, blue – blood in the perianal region, red – rectal prolapse, granulation, fistula. After week 9 no more mice were lost and the signs of inflammation gradually regressed.

*
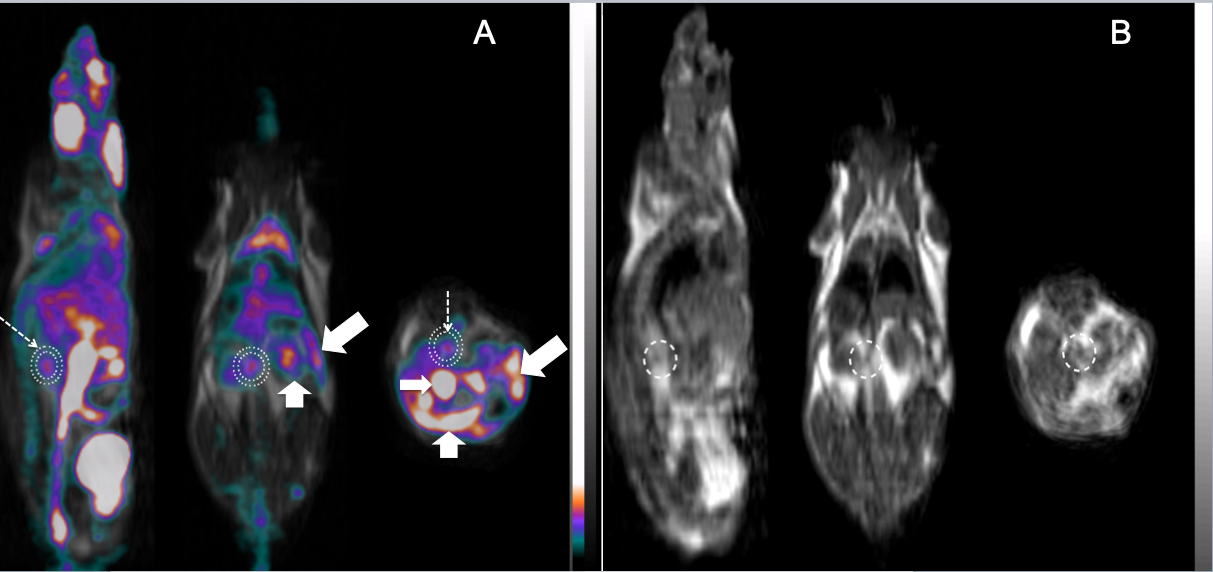
*


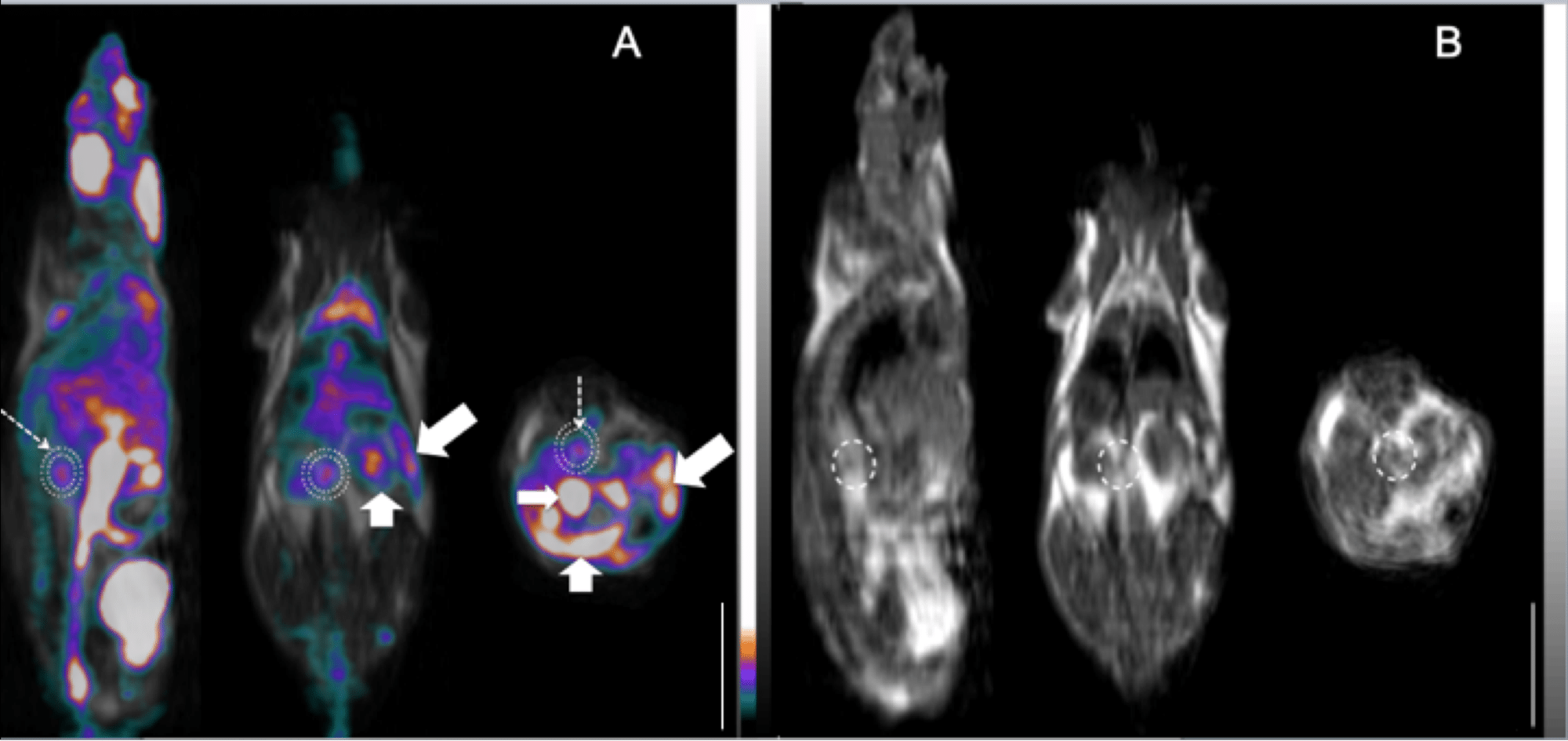


*Figure S4: MRI and PET imaging, showing an enlarged retroperitoneal lymph node in MRI, with water signal enhancements and its moderate-high FDG uptake in a WT animal.* The same retroperitoneal lymph node with edematous infiltration is shown in the MR images with a dashed circle. Both panels show similarly ordered 2D sections, sagittal (left), horizontal (mid) and cross-sectional (right) planes of the animals. Scale bars indicate 500 microns. Color scale indicates PET signal intensity, greyscale indicates MRI signal intensity.


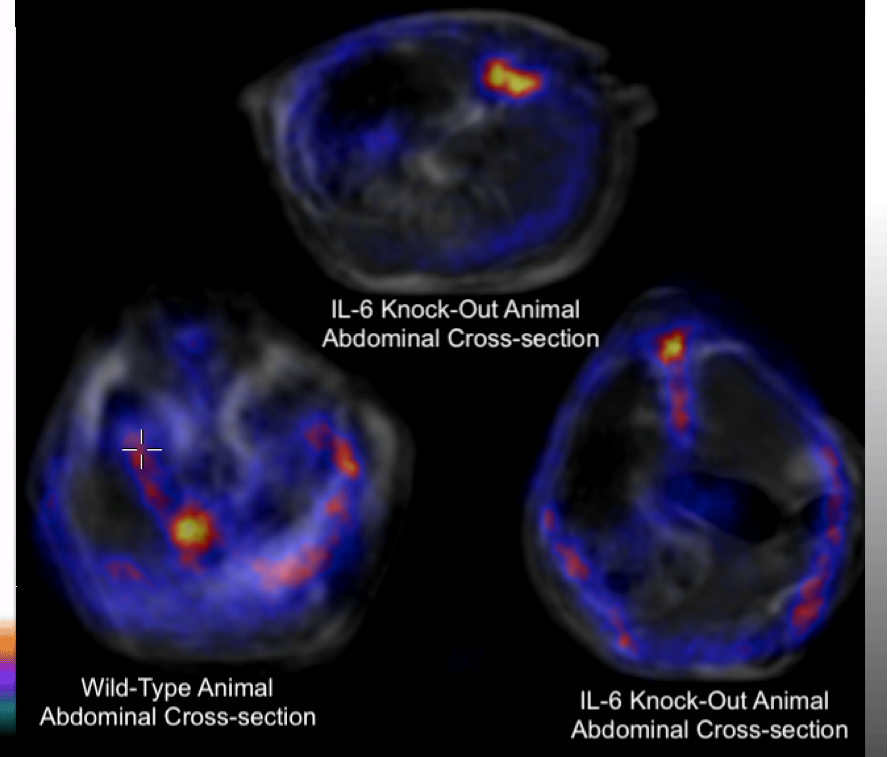


*Figure S5: ^18^F-FDG PET/MRI image of two IL-6 KO and one WT animals in an abdominal cross-sectional plane, placed in the multi-animal mouse holder bed*. The hair cross shows the accumulation of ^18^F-FDG in the wall of the colon. The simultaneous placement of mice in the multi-animal bed enabled to create an abdominal cross-sectional overview of the reconstructed PET/MRI images. Color scale indicates PET signal intensity, greyscale indicates MRI signal intensity.

| WT SUVmax values  [g/mL]  Mean: 21.4 (8.0) | IL-6 KO 1 SUVmax values  [g/mL]  Mean:6.5 (3.2) | IL-6 KO 2 SUVmax values  [g/mL]  Mean: 5.5 (3.0) |
| --- | --- | --- |
| 25.3 | 8.2 | 3.4 |
| 26.8 | 8.4 | 8.9 |
| 12.2 | 2.8 | 4.2 |

*Supplementary Table 1: ^18^F-FDG uptake comparison in the highly avid tumorous foci in the WT animal and the two IL-6 KO animals.* Regional Maximal Standardized Uptake Values (SUVmax) are presented, to allow assessment of tumor glycolytic activity comparison. The three most FDG-avid focal regions in the infradiaphragmatic area have been selected in Interview Fusion (Mediso, Hungary) software in each mouse PET image and their proportionated SUVmax values are compared in the table. Standard deviation is shown in parentheses after the mean.
