# Supplementary figures and images for: Thrombocytosis and Effects of IL-6 Knock-Out in a Colitis-Associated Cancer Model
Source: Int J Mol Sci. 2020 Aug 27;21(17):6218. doi: 10.3390/ijms21176218 (PMC7504541; doi:10.3390/ijms21176218)

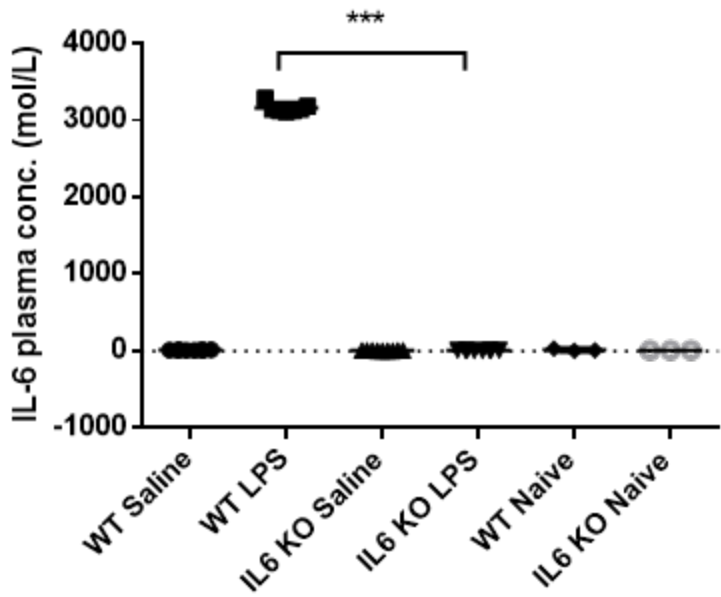

Supplement: Supplementary file 1 [file ijms-21-06218-s001.zip › ijms-874497 supplementary/Uploading supplementary/Fig S1.pdf]

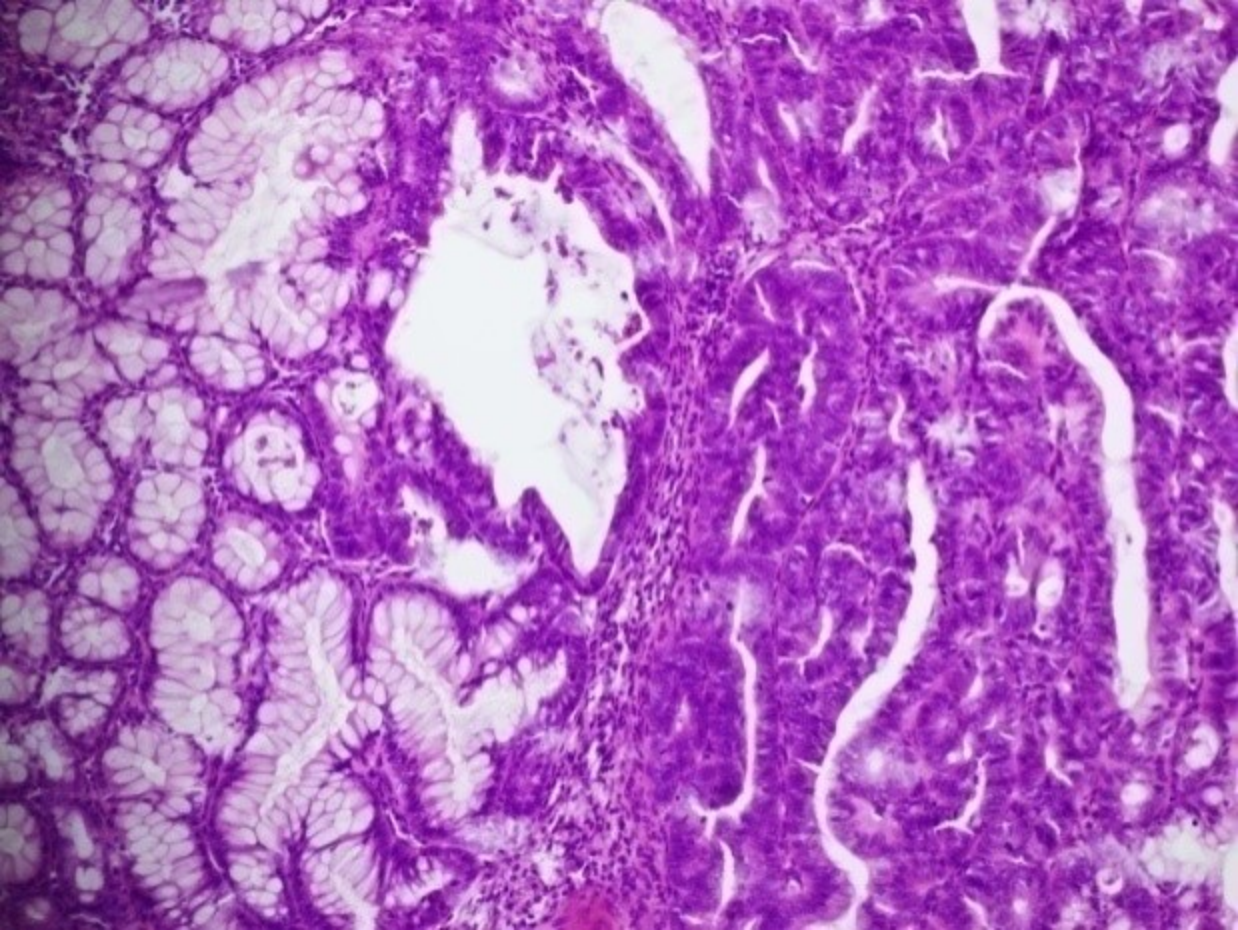

Supplement: Supplementary file 1 [file ijms-21-06218-s001.zip › ijms-874497 supplementary/Uploading supplementary/Fig S2.pdf]

# IL-6 KO

SCORE

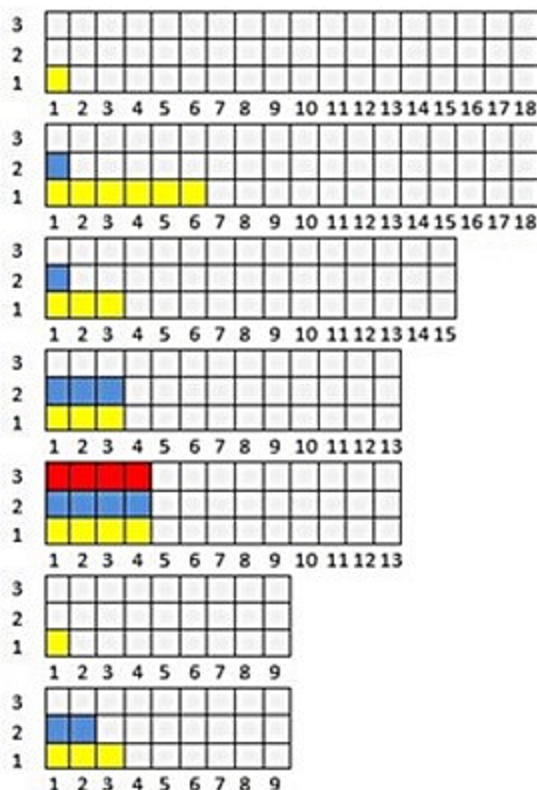

# WT

Day 3

Week 1

Week 3

Week 5

Week 6

Week 7

Week 9

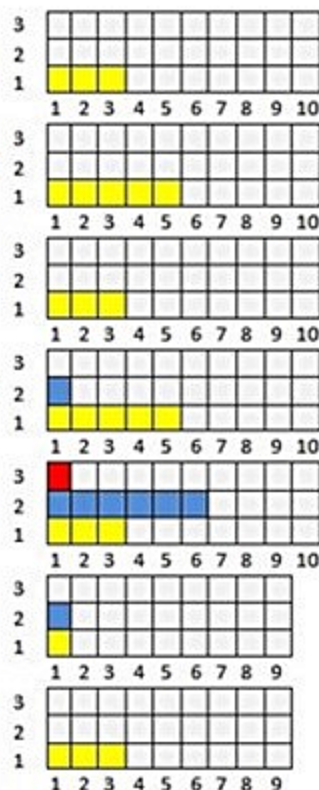

Number of mice

Supplement: Supplementary file 1 [file ijms-21-06218-s001.zip › ijms-874497 supplementary/Uploading supplementary/FigS3.pdf]

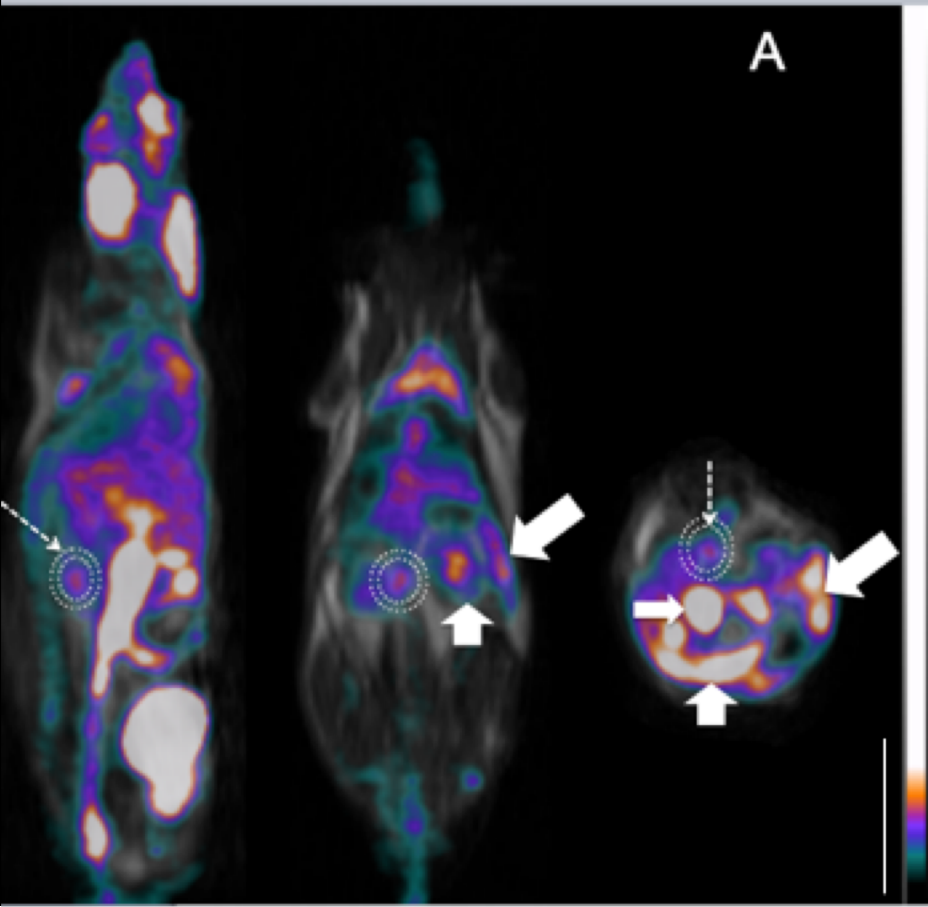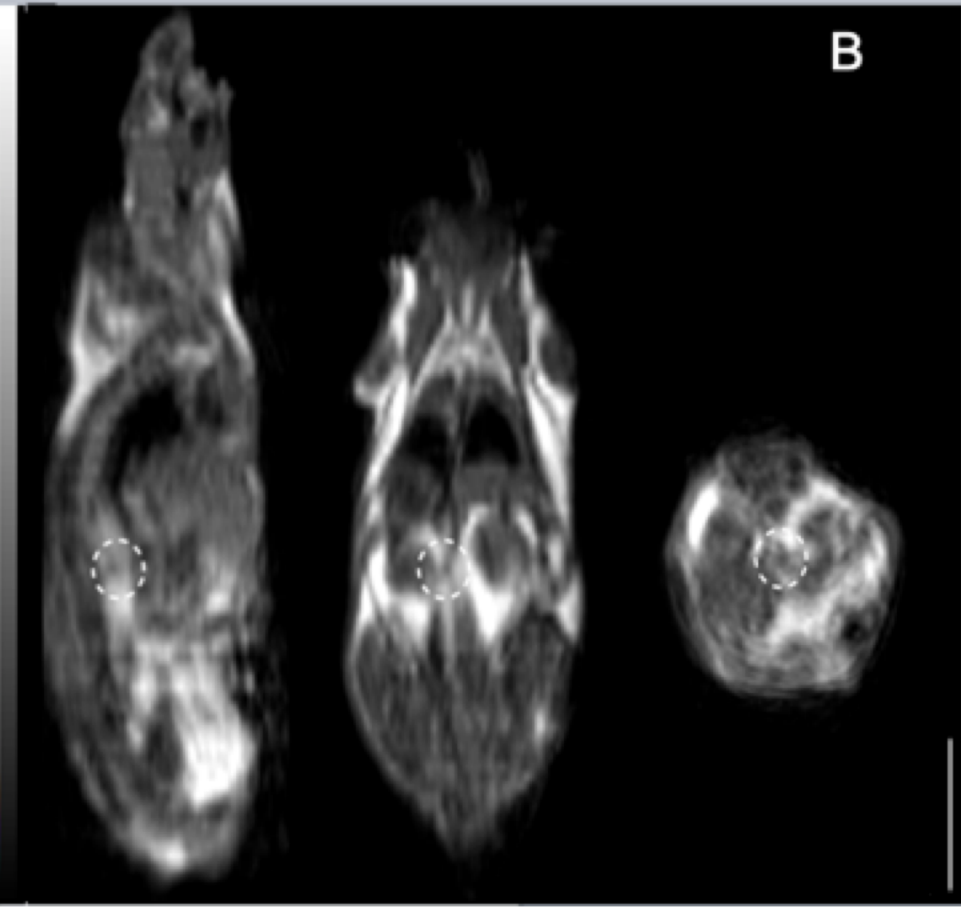

Supplement: Supplementary file 1 [file ijms-21-06218-s001.zip › ijms-874497 supplementary/Uploading supplementary/FigS4_scalebar.pdf]
